# The Treatment of Dacryocystitis

**Published:** 1904-05-14

**Authors:** 


### THE TREATMENT OF DACRYOCYSTITIS.

A plea for a more radical plan than is usually adopted in treating chronic inflammatory conditions of the lachrymal sac is put forward by Dr. A. Wiener.[1] In support of his contention he urges that a chronic blenorrhœa of the sac provides a constant source of infection which may result in great damage to the eye, or even lead to its destruction. He therefore advises that if the usual methods, such as passage of probes to dilate stricture of the nasal duct, intra-nasal treatment to free the lower opening of the duct, and so forth, be unavailing after a fairly patient trial, the best thing to do is to extirpate the lachrymal sac, an operation which may usefully be performed also in some cases which have not yet become chronic. Dr. Wiener lays down the following indications for the operation :—(1) In fistula of the lachrymal sac after long-continued suppuration (acute cases excepted). (2) In chronic blenorrhœa of the sac. (3) In chronic catarrh of the sac with an organic stricture. (4) In chronic blenorrhœa when disease of the bone with caries is present. (5) In relatively fresh cases of blenorrhœa, under the following conditions : (*a*) In ulcus serpens, when the healing is very much favoured by an immediate extirpation of the sac ; (*b*) when an operation for cataract or glaucoma is contemplated ; (*c*) in fresh injuries of the cornea. (6) Tuberculosis of the sac.

The operation is not difficult and does not require the administration of a general anæsthetic, the local use of cocaine and adrenalin being sufficient. A semi-lunar incision is made two and a half centimetres in length and about half a centimetre from the inner canthus of the eye, the incision commencing just above the internal palpebral ligaments. The sac is exposed, and if possible removed without being opened, the dissection being carried out with dressing forceps and scissors. Healing will be complete by the seventh day in a typical case, and after three weeks it will be nearly impossible to detect the scar. It is most important to remove the whole of the sac, for if a small piece be left behind the wound will not heal. With regard to after-effects, Dr. Wiener states that there is nothing to say against the operation. The resulting epiphora is not as a rule very annoying to the patient, and is amply compensated for by the removal of a constant menace to the affected eye.

[1] Med. Rec., April 2.

